# List of reviewers

**DOI:** 10.2471/BLT.25.970125

**Published:** 2025-01-01

The Editorial Team would like to thank all those who gave generously of their time and expertise in reviewing the papers for the *Bulletin of the World Health Organization *in 2024. We apologize to anyone whose name has been inadvertently omitted from the following list:

## A

Abo-Rahma, Alyaa

Abu Sin, Muna

Adebajo, Adewale

Ade-Banjo, Olympus

Adedeji, Waheed

Adesina, Adebiyi

Adnan, Hassan Sami

Aduo-Adjei, Kofi

Akin, Ayse

Alam, Muhammed Ashraful

Alkhawaja, Safa

Allen, Koya

Allen, James

Al-Naggar, Redhwan

Aloosh, Mehdi

Alveano, Jesus

Amer, Faten

Anaele, Beverly

Ancker, Jessica

Anderson, Ian

Anderson, Michelle

Ansah, Edward

Aqil, Anwer

Arbon, Paul

Armand, Alex

Arnold, Laura

Asefa, Nigus

Ashour, Hossam

Avoka, Cephas

Ayala, Roberto

## B

Başar, Dilek

Backholer, Kathryn

Badr, Kamal

Baek, Yeji

Bailey, Helen

Barber, Sarah

Bassett, Ken

Bates, David

Benova, Lenka

Beran, David

Berer, Marge

Best, Emma

Bhaskaranand, Malavika

Bhatti, Aikant

Bhushan, Indu

Bianchini, Alahí

Birdthistle, Isolde

Bisrat, Dawit

Blanchard, Laurence

Blando, James

Blencowe, Hannah

Boci, Arian

Boerma, Ties

Borst, Robert

Bose, Bijetri

Botabara-Yap, Mary Jane

Boydell, Vicky

Branca, Francesco

Brewer, Timothy

Brisson, Julien

Brommels, Mats

Brummer, Lukas

## C

Campos, Josefina

Candeias, Vanessa

Capewell, Simon

Capraro, Valerio

Carden, Fred

Carmona, Loreto

Castellanos, Erika

Castro Lopes, Sofia

Cato, Susumu

Causevic, Sara

Celik, Sebahattin

Chang, Andrew

Changula, Katendi

Chapman, Jeremy

Chaurasia, Akhilanand

Chavarria Pino, Eliana

Chen, Guochong

Christensen, Rene

Christofield, Megan

Cieza, Alarcos

Claassen, Cassidy

Clapp, Jennifer

Clark, Joseph

Codyre, Patricia

Coetzee, David

Conigrave, Katherine

Corvalan, Carlos

Coulibaly, Karna

Crocker-Buque, Tim

Cronk, Ryan

Crowcroft, Natasha

Cull, Benjamin

Curioso, Walter

## D

D'Agostino, Marcelo

Daley, Matthew

Dalugoda, Thudawe

Damor, Raman

Danis, Marion

Darney, Blair

Das, Saibal

Datta, Manjula

Davoli, Marina

De Villiers, Melgardt

Deboutte, Danielle

Delamalle, Jean-Pierre

Demarzo, Marcelo

Devine, Alexandra

Dol, Justine

Dolk, Helen

Domínguez, Angela

Donoho, Daniel

Duggal, Mona

Durrheim, David

## E

Ebi, Kristie

Eide, Arne

Emeje, Martins

Esiet, Uwem

## F

Fan, ZhiXin

Farooqi, Najiha

Fatima, Anab

Faubion, Stephanie

Ferenchick, Erin

Ferreira, Marcelo

Figueras, Albert

Fisher, Jane

Flici, Farid

Forde, Andrea

Forrester, Terrence

Forzley, Michele

Fraim, Nalan

Fraser, Joy

## G

Gable, Lance

Gajewski, Byron

García-Moreno, Claudia

García Valiña, Luis 

Gebeyehu, Natnael

Geneau, Robert

Ghasemi, Hadi

Gkioka, Vasiliki

Graham, Cynthia

Gray, Andrew

Greenhalgh, Trish

Grigorovich, Alisa

Grilo, Graziele

Gu, Jing

Guidos, Brittany

Guthold, Regina

Gutiérrez Martínez, Anthony

## H

HabibiSaravi, Reza

Hadler, Maria Claret

Hakeem, Rubina

Halsey, Eric

Hanifah, Laily

Haq, Zaeem

Haque, Rabiul

Hartsough, Kieran

Hasman, Andreas

He, Jiawei

Heath, Paul

Hedayati, Seyed Pouria

Hertzog, Lucas

Hodgins, Stephen

Hogerzeil, Hans

Holmes, Wendy

Honda, Tomoko

Howell, Jessica

Huttner, Benedikt

## I

Ikuomola, Felix

## J

Jaishwal, Chandni

Jamshidi, Hamidreza

Jayatilleke, Achini

Jenei, Kristina

Jhaveri, Ravi

Jiang, Hongbo

Jin, Hui Min

Johri, Mira

Joseph, K

Joshi, Niravkumar

## K

Kågesten, Anna

Kaindama, Lukeki

Kakade, Saurabh

Kamalakannan , Sureshkumar

Kamenov, Kaloyan

Karanikolos, Marina

Karthickeyan, DSA

Kassam, Dilshad

Kaur, Ismeet

Kazemi, Samiyeh

Khatri, Resham

Khorrami, Zahra

Kim, Honghyok

Kinder, Karen

Kindrachuk, Jason

King, Carina

Kita, Keiko

Kohlmann, Sebastian

## L

Labrique, Alain

Lackland, Daniel

Lahariya, Chandrakant

Lambert, Pete

Land, Mary-Anne

Lara Lona, Elia

Laslett, Anne-Marie

Lau, Eric

Leandowsky, Stephan

Lei, Dianliang

Leung, Kathy

Lewis, Rosamund

Leye, Dessalegn Temesgen

Li, Wenzhen

Liaw, Siaw-Teng

Liu, Dianchang

Lloyd-Sherlock, Peter

Lopert, Ruth

Lopes, Garrafa Julia

López-Encuentra, Ángel

Lopez-Hernandez, Daniel

Lotfizadeh, Masoud

Lubis, Inke

Lunt, Neil

Luthra, Rita

Lythgoe, Mark

## M

Ma, Yan

Maaß, Laura

Mabiama, Gustave

Mahendra, Shanti

Maheu-Giroux, Mathieu

Makali, Samuel

Mammo, Tihitena Negussie

Mann, J. John

Mantel-Teeuwisse, Aukje

Maravilla, Joemer

Marchant, Tanya

Mariotti, Silvio

Marshall, Brandon

Mastroleo, Ignacio

Mathur, Arvind

Mathur, Roli

Matiwane, Busisiwe

Matzopoulos, Richard

McCrindle, Brian

Mehmood, Tahir

Menon, Jaideep

Mestrovic, Tomislav

Minh Khue, Pham

Mire, Amina

Mitchell, Rob

Mittan, Sandeep

Mohammad, Yousser

Montgomery, Maggie

Morales Ruiz, Paulina

Morand, Serge

Moroz, Paul

Mousally, Jon

Mudzviti, Tinashe

Mullen, Lucia

Mulyadi, Asal Wahyuni Erlin

Myrttinen, Henri

## N

Narayan, Choudhary

Neffati, Larbi

Newell-Jones, Katy

Newton, Paul

Nicholson, Emily

Nihlen, Asa

Noori, Teymur

Norris, Susan

Nugent, Rachel

## O

Olawumi, Abdulgafar

Olusanya, Bolajoko

Opoku, Daniel

Orenstein, Walter

Orkin, Aaron

Orlien, Stian

Oruc, Yesim

Owusu-Boaitey, Nana

Oyediran, Kolawole

## P

Pandya, Apurvakumar

Pannenborg, Ok

Papanicolas, Irene

Parker, Richard

Patel, Mahomed

Patel, Jay

Patel, Nirav

Patterson, David

Paul, Elisabeth

Paunio, Mikko

Pecoul, Bernard

Pereira, Maísa

Perera, Sathira

Perucic, Anne'Marie

Petkovic, Jennifer

Phelps, Benjamin

Phillips, J. Craig

Philpott, Anne

Pintye, Jillian

Pitman, Alexandra

Piyasena, Mapa Prabhath

Plata Casas, Laura

Prabhune, Akash

## Q

Quddus, Arshad

Quigley, Paula

## R

Raghunath Rao, Rangasayee

Rahman, Mashiur

Rahman, Mizanur

Rajan, Dheepa

Rayner, Michael

Reynolds, Nancy

Richards, Nicola

Rikap, Cecilia

Rizzi, Menico

Rodewald, Lance

Rohloff, Peter

Romero, Carmen

Rosenberg, Nora

Rubeis, Giovanni

## S

Sabet, Farnaz

Salama, Khaled

Saleh, Mai

Sarti, Flavia

Saunders, Philippa

Savadogo, Abdou-Salam

Saxena, Sangeeta

Scarpino, Samuel

Schade, Alexander

Schultz, Sally

Schwalbe, Nina

Sconza, Rebecca

Scott, Kerry

Seijas, Vanessa

Selleck, Paul

Sengupta, Amit

Shah, Sanjeeb

Shah, AMM Mubassher

Shakespeare, Tom

Shankar, P Ravi

Sharfstein, Joshua

Sharma, Priya

Sharma, Arvind

Sharma, Reet

Sharma, Tarang

Shringarpure, Kalpita

Shulman, Lawrence

Siegel, Carole

Silva, Angélica

Silva, Ricardo

Singh, Satendra

Siswati, Tri

Smith, Julie

Solerdelcoll Arimany, Mireia

Solomon, Daniel

Steel, Robert

Stoto, Michael

Struminger, Bruce

Sturchio, Jeffrey

Suelves, Josep M

Sule, Salami

Suleman, Qazi

Suraweera, Wilson

Susilawati, Sri

Sy, Deborah

Sy, Tyrone

## T

Tan, Kean Chye

Tang, Yi

Tanveer, Sandila

tenBrink, Debra

Terrones-Lozano, Alejandro

Thapa, Rajshree

Tholandi, Maya

Thomas, Rebekah

Thompson, Peyton

Thulaseedharan, Jissa

Tikkanen, Roosa

Tong, Deborah

Toni, Esmaeel

Traore, Tieble

Tsai, Jack

Tshokey, Tshokey

Tucker, Joseph

Tully, Kristin

Turbow, Sara

Turner, Tari

## V

van den Akker, Thomas

van der Eijk, Yvette

Vater, Maria

Veillard, Jeremy

Veltri, Giuseppe

Vervoort, Dominique

Vlaev, Ivo

Vogel, Thomas

Voyi, Kuku

## W

Wahdi, Amirah

Walson, Judd

Wang, Quanyi

Wang, Xin

Waruingi, Daniel

Waterworth, Christopher

Weis, Julianne

Wieringa, Frank

Wilder-Smith, Petra

Willcox, Merlin

Wright, Katherine

## Y

Yeager, Beth

Yeatman, Sara

Yuvaraj, Anandi

## Z

Zhang, Li

Zhou, Suizan

Ziafati Bafarasat, Abbas

Zimmermann, Bettina

Zinsstag, Jakob

Zou, Zhiyong

Zuo, Genyong

Zwijnenburg, Jorrit

